# Supplementary material for: The Systems Biology Research Tool: evolvable open-source software
Source: BMC Syst Biol. 2008 Jun 29;2:55. doi: 10.1186/1752-0509-2-55 (PMC2446383; doi:10.1186/1752-0509-2-55)
Supplement: Additional file 1 — SBRT Archive. An archive of the current version of the Systems Biology Research Tool. [file 1752-0509-2-55-S1.zip › sbrt-1.4.0/doc/developers_guide/api/index-files/index-22.html]

V-Index


|  |  |  |
| --- | --- | --- |
| **Overview** \| Package \| Class \| Use \| **Tree** \| **Deprecated** \| **Index** \| **Help** | |  |
| **PREV LETTER**   **NEXT LETTER** | **FRAMES**    **NO FRAMES**     **All Classes** |  |


A B C D E F G H I J K L M N O P Q R S T U V W X Y Z 

---


## **V**

**valueOf(String)** - Static method in enum sbrt.kernel.math.optimization.OptimizationSense: Returns the enum constant of this type with the specified name. **valueOf(String)** - Static method in enum sbrt.kernel.math.optimization.ProgramSolverStatus: Returns the enum constant of this type with the specified name. **values()** - Static method in enum sbrt.kernel.math.optimization.OptimizationSense: Returns an array containing the constants of this enum type, in the order they're declared. **values()** - Static method in enum sbrt.kernel.math.optimization.ProgramSolverStatus: Returns an array containing the constants of this enum type, in the order they're declared. **values()** - Method in class sbrt.kernel.util.UnmodifiableMap: See Map.values(). **VariableParticipation**<E extends MathExpr<V>,V> - Class in sbrt.kernel.utilities.proc: This class is used to organize a collection of mathematical expressions according to the variables they contain. **VariableParticipation()** - Constructor for class sbrt.kernel.utilities.proc.VariableParticipation: **VariableParticipationManager** - Class in sbrt.shell.mng.utilities: This class is used to manage variable participation experiments. **VariableParticipationManager()** - Constructor for class sbrt.shell.mng.utilities.VariableParticipationManager: **variables()** - Method in class sbrt.kernel.math.AbstractLinearComb.Term: Returns `1`. **variables()** - Method in class sbrt.kernel.math.algebra.HashLinearSystemSolution: Returns the total number of variables in this solution. **variables()** - Method in interface sbrt.kernel.math.algebra.LinearSystemSolution: Returns the total number of variables in this solution. **variables()** - Method in class sbrt.kernel.math.HashLinearComb: Returns the number of variables in this mathematical expression. **variables()** - Method in interface sbrt.kernel.math.MathExpr: Returns the number of variables in this mathematical expression. **variables()** - Method in interface sbrt.kernel.math.Polynomial.Term: Returns the number of variables contained in this term. **VectorComparer** - Class in sbrt.kernel.math: This class is used to compare vectors of double precision numbers. **VectorComparer()** - Constructor for class sbrt.kernel.math.VectorComparer: **VectorComparer.Diff**<V> - Class in sbrt.kernel.math: Instances of this class are used to hold a variable and a pair of corresponding intervals. **VectorComparison**<V> - Class in sbrt.kernel.utilities.proc: This class is used to compare sets of intervals. **VectorComparison()** - Constructor for class sbrt.kernel.utilities.proc.VectorComparison: **VectorComparisonManager** - Class in sbrt.shell.mng.utilities: This class is used to compare the intervals contained in separate files. **VectorComparisonManager()** - Constructor for class sbrt.shell.mng.utilities.VectorComparisonManager: **VectorIntersection**<K,V> - Class in sbrt.kernel.math.algebra.proc: This class is used to intersect vectors whose variables (that is, map keys) are not identical. **VectorIntersection()** - Constructor for class sbrt.kernel.math.algebra.proc.VectorIntersection: Constructs a new vector intersector. **VERSION\_ARG** - Static variable in class sbrt.Sbrt: The argument used to display the version number.

---


|  |  |  |
| --- | --- | --- |
| **Overview** \| Package \| Class \| Use \| **Tree** \| **Deprecated** \| **Index** \| **Help** | |  |
| **PREV LETTER**   **NEXT LETTER** | **FRAMES**    **NO FRAMES**     **All Classes** |  |


A B C D E F G H I J K L M N O P Q R S T U V W X Y Z 

---
